# Artificial intelligence‐assisted analysis of musculoskeletal imaging—A narrative review of the current state of machine learning models

**DOI:** 10.1002/ksa.12702

**Published:** 2025-06-01

**Authors:** Felix C. Oettl, Bálint Zsidai, Jacob F. Oeding, Michael T. Hirschmann, Robert Feldt, David Fendrich, Matthew J. Kraeutler, Philipp W. Winkler, Pawel Szaro, Kristian Samuelsson, ESSKA Artificial Intelligence Working Group

**Affiliations:** ^1^ Department of Orthopedic Surgery Balgrist University Hospital University of Zürich Zurich Switzerland; ^2^ Hospital for Special Surgery New York New York USA; ^3^ Department of Orthopaedics Institute of Clinical Sciences, Sahlgrenska Academy University of Gothenburg Gothenburg Sweden; ^4^ Sahlgrenska Sports Medicine Center Göteborg Sweden; ^5^ Department of Orthopaedic Surgery and Traumatology Kantonsspital Baselland Bruderholz Switzerland; ^6^ University of Basel Basel Switzerland; ^7^ Department of Computer Science and Engineering Chalmers University of Technology Gothenburg Sweden; ^8^ Tenfifty Gothenburg Sweden; ^9^ Department of Orthopaedic Surgery & Rehabilitation Texas Tech University Health Sciences Center Lubbock Texas USA; ^10^ Department for Orthopaedics and Traumatology Kepler University Hospital GmbH, Johannes Kepler University Linz Linz Austria; ^11^ Department of Radiology Sahlgrenska Academy University of Gothenburg Gothenburg Sweden; ^12^ Department of Orthopaedics Sahlgrenska University Hospital Gothenburg Sweden

**Keywords:** AI in musculoskeletal imaging, clinical integration, computer vision, deep learning, image analysis

## Abstract

The potential of Artificial intelligence (AI) is increasingly recognized in musculoskeletal radiology, offering solutions to challenges posed by increasing imaging volumes and fellowship trained radiologist shortages. The integration of AI is not intended to replace radiologists but to augment their capabilities, improving workflow efficiency and diagnostic accuracy. This narrative review examines the current landscape of AI applications in musculoskeletal imaging, focusing on both general‐purpose multimodal models and specialized foundation models. AI has proven effective in musculoskeletal imaging, enhancing fracture detection, scoliosis assessment, and lower limb alignment analysis. In osteoarthritis, AI aids early detection by identifying subtle structural changes. AI‐accelerated MRI reconstruction reduces scan times by up to 90% while maintaining diagnostic quality, improving efficiency and accessibility. Emerging multimodal models further integrate imaging with clinical data, advancing precision medicine. Technical challenges persist, particularly in addressing motion artifacts and anatomical complexity. Ethical considerations, including data privacy, algorithmic bias, and model transparency, remain crucial for responsible implementation. While challenges remain in clinical validation and implementation, the combination of broad and narrow AI models shows promise in advancing precision medicine and democratizing quality care.

**Level of Evidence:**

Level V.

**Abbreviations**
AI: artificial intelligence
CT: computer tomography
MRI: magnetic resonance imaging
OA: osteoarthritis
SAM: segment anything model

## INTRODUCTION

Artificial intelligence (AI) has emerged as a powerful tool in medical imaging, including musculoskeletal radiology [44, 49]. By leveraging advanced algorithms and big data, AI enables the automation of image analysis, offering the potential to improve diagnostic accuracy, reproducibility and efficiency [9, 15, 24]. This is especially valuable in improving patient flow and reducing turnover times in musculoskeletal imaging, where the interpretation of radiographs, computed tomography (CT), and magnetic resonance imaging (MRI) scans, can be intricate and time‐consuming.

Considering increasing imaging volumes and a relative shortage of subspecialized musculoskeletal radiologists, AI offers the potential for a scalable solution, democratising accurate diagnosis and therefore quality of care. By triaging imaging examinations and assisting with image interpretation, AI‐based tools furthermore reduce reporting times. The integration of AI into clinical practice is not intended to replace radiologists but to augment their capabilities, allowing them to manage increasing workloads effectively [12].

While AI has demonstrated significant potential in musculoskeletal imaging, its clinical adoption requires validation, ethical oversight, and consideration of its limitations. This review aims to explore these aspects to provide a balanced perspective on the future of AI in musculoskeletal radiology including exploration of the types of AI models utilised, ethical considerations, technical challenges, and current solutions.

## TYPES OF AI MODELS IN MEDICAL IMAGE ANALYSIS

### Broad machine learning (ML) models

Multimodal AI models such as Gemini, ChatGPT, Llama and Claude have the potential to disrupt medical image analysis through their ability to process both visual and textual data. These models can integrate visual data from medical imaging with clinical information from the patient's electronic health record, enabling comprehensive diagnostic assessments across multiple medical domains [37]. For instance, the Llama 3.2‐90B model demonstrated superior performance in 85.27% of cases, outperforming human experts on assessment of CT reports [37].

These multimodal models exhibit great versatility in musculoskeletal imaging, supporting complex tasks such as fracture detection, bone age assessment, osteoarthritis grading and tumour diagnosis [11, 26]. Emerging technologies including Google's Med‐PaLM are expanding these capabilities, enabling more sophisticated interpretation of biomedical information [6, 45, 49]. However, current limitations such as potential biases, occasional diagnostic inaccuracies and the need for extensive validation across diverse clinical populations hold back widespread adoption. Ongoing research focuses on improving model reliability, reducing algorithmic bias, and developing robust human‐AI collaborative diagnostic frameworks [37, 45].

### Narrow ML models

Narrow ML models are tailored to specific domains including natural language processing and computer vision [50]. These models are meticulously trained on domain‐specific data sets, enabling them to perform tasks within narrow fields with remarkable precision.

Two examples illustrate the potential of Narrow AI. The Segment Anything Model (SAM), developed by Meta AI, represented a breakthrough in image segmentation, demonstrating remarkable versatility across multiple imaging domains [19]. Similarly, GE HealthCare's radiograph model showcases the targeted potential of these models, providing specialised capabilities for medical imaging analysis. These models are typically constructed through pre‐training on extensive domain‐specific datasets, adapting existing architectures like BERT and Swin, and incorporating multispectral data handling techniques.

One subgroup, Specialized Foundational Models, are of a special area of particular interest, however research indicates that such models have not consistently outperformed smaller supervised models, suggesting the field remains in a state of development [50]. Their primary characteristics include domain‐specific training, high adaptability, transfer learning capabilities, and significant scalability, though they demand substantial computational resources [50].

Broad AI models, approaching a primitive general AI, represent a versatile approach to healthcare, including medical imaging, distinguished by their ability to handle tasks across multiple imaging modalities [30]. These models leverage big data, enabling transfer learning and adaptation to emerging medical challenges [30, 47]. Their architecture allows for analysis across medical specialties, however, this broad approach is not without limitations—as these models often struggle with domain‐specific nuances and require substantial computational infrastructure for effective training and deployment [30]. Narrow AI models, in contrast, offer precision‐engineered solutions focused on e.g. specific medical imaging subspecialties. By integrating deep, domain‐specific knowledge and training on targeted datasets, these models can achieve superior accuracy in niche diagnostic tasks [43]. They excel in areas requiring intricate 'understanding', such as rare disease detection and precise anatomical segmentation. Their primary constraint remains their limited generalisability outside of the specific trained domain, necessitating multiple specialized models for comprehensive medical imaging workflows [43].

The future of medical image analysis lies in integration strategies that synthesise the strengths of both broad and narrow models. Emerging approaches include hybrid model architectures, advanced transfer learning techniques, ensemble methodologies, and federated learning frameworks [2, 30, 43, 47]. These strategies aim to create more robust, adaptable, and interpretable AI systems that can augment clinical decision‐making while maintaining high diagnostic accuracy and reliability. Researchers and clinicians recognised that the most promising path forward is not a binary choice between broad and narrow models, but a collaborative approach that leverages the strengths of multiple models. By developing context‐aware AI frameworks that can dynamically adapt and specialise, healthcare providers can unlock unprecedented potential in diagnostic imaging, ultimately improving patient outcomes and advancing precision medicine [24, 30].

## TECHNICAL CHALLENGES IN MUSCULOSKELETAL IMAGING AI

### Image quality

Motion artefacts represent a critical challenge in musculoskeletal imaging, particularly in imaging systems like EOS imaging used for spinal pathology diagnostics. With acquisition times extending up to 25 seconds, patient movement can introduce significant image distortions that challenge accurate diagnostic interpretation [46]. Researchers developed a systematic approach to quantify these artifacts by attaching a radiopaque reference device (a straight metal wire) to patients and measuring deviations from a precise vertical line. They found that 80% of patients demonstrated motion artifacts exceeding 1 mm in frontal projection, while 87.9% showed similar artefact levels in lateral projections [46]. These high artefact rates underscore the critical need for AI algorithms capable of distinguishing between genuine pathological findings and image distortions caused by patient movement. The potential for motion artifacts to mimic conditions like scoliosis highlights the complexity of developing robust medical imaging analysis technologies that can maintain diagnostic accuracy under challenging imaging conditions.

In a 2024 study, researchers evaluated an AI‐based bone scan noise‐reduction filter for whole‐body planar bone scintigraphy [8]. The filter demonstrated promising capabilities, enhancing image quality and contrast while allowing a potential 50% reduction in administered dose or acquisition time. By successfully processing artificially degraded noisy images with varying total count levels, the AI filter significantly improved diagnostic confidence in low‐count imaging scenarios.

Deep learning image reconstruction, an entirely different field of research, has made substantial advances in noise reduction across medical imaging modalities. A 2024 study revealed that AI‐based reconstruction techniques enable 60% accelerated volumetric brain MRI while preserving quantitative performance, with similar improvements observed in spinal MRI scans in which 40% faster scans matched or exceeded standard care quality [4, 5].

### Addressing the complexity of the musculoskeletal system

The anatomical complexity of the musculoskeletal system presents challenges for AI in medical imaging, particularly in structure identification. Foundation models like SAM, MedSAM, and SAM2 are being evaluated for processing diverse anatomical structures in musculoskeletal MRI, while AI‐powered ultrasound systems such as Clarius MSK AI can now identify and label key anatomical structures in real time [14]. However, segmentation of musculoskeletal structures remains complex, with deep learning models and convolutional neural networks (CNNs) (e.g., TotalSegmentor) showing potential [12, 48]. Researchers including Liu et al. have successfully used advanced modelling techniques to accurately segment knee structures, including cartilage, menisci, and bones [25]. However, challenges persist in segmenting structures with low contrast or those affected by pathological changes, driving ongoing research to improve algorithmic accuracy and robustness [11]. Additionally, the potential of AI‐assisted image segmentation relies heavily on the unbiased ability of models to accurately detect bony morphology and soft‐tissue structures among patients with different ages, sex, and racial backgrounds. Recent research discusses potential strategies to mitigate bias in automated image segmentation through computational methods that may enhance fairness based on certain tradeoffs in the applied modelling approach [41].

Mapping relationships between musculoskeletal structures is crucial for understanding biomechanics and pathology. Emerging techniques include joint‐muscle mapping using neural networks, three‐dimensional imaging reconstructions, and algorithms for image registration [18, 44]. These approaches aim to analyse spatial configurations, express nonlinear relationships between joint angles and muscle lengths, and facilitate more comprehensive understanding of complex anatomical interactions.

## AI SOLUTIONS AND APPLICATIONS

AI has been promising in accurately detecting fractures on imaging, enhancing triage, diagnosis, and patient care, particularly in emergency settings [33]. A systematic review and meta‐analysis of 100 studies reported AI algorithms achieving a sensitivity of 91.43% and specificity of 92.12% for fracture detection on plain radiographs, highlighting their potential as valuable tools for clinicians [33]. In a multi‐reader, multi‐case study, nonspecialist readers using AI support improved their patient‐wise sensitivity from 72% to 80% and specificity from 81% to 85%, resulting in a 29% relative reduction in missed fractures [3]. Key benefits of AI integration include improved diagnostic accuracy, faster interpretation times, support for less experienced clinicians, triage assistance for prioritising urgent cases, and standardised assessments that reduce interobserver variability [3, 28, 40]. Recently the UK's National Institute for Health and Care Excellence has endorsed AI technologies for fracture detection, recognising their ability to improve diagnostic performance in urgent care compared to standard care alone [32]. As these tools are designed to assist rather than replace human clinicians, their highest performance is achieved when used alongside expert interpretation, underscoring the importance of ongoing research and clinical validation to optimise their role in musculoskeletal imaging [15].

**Figure 1:**
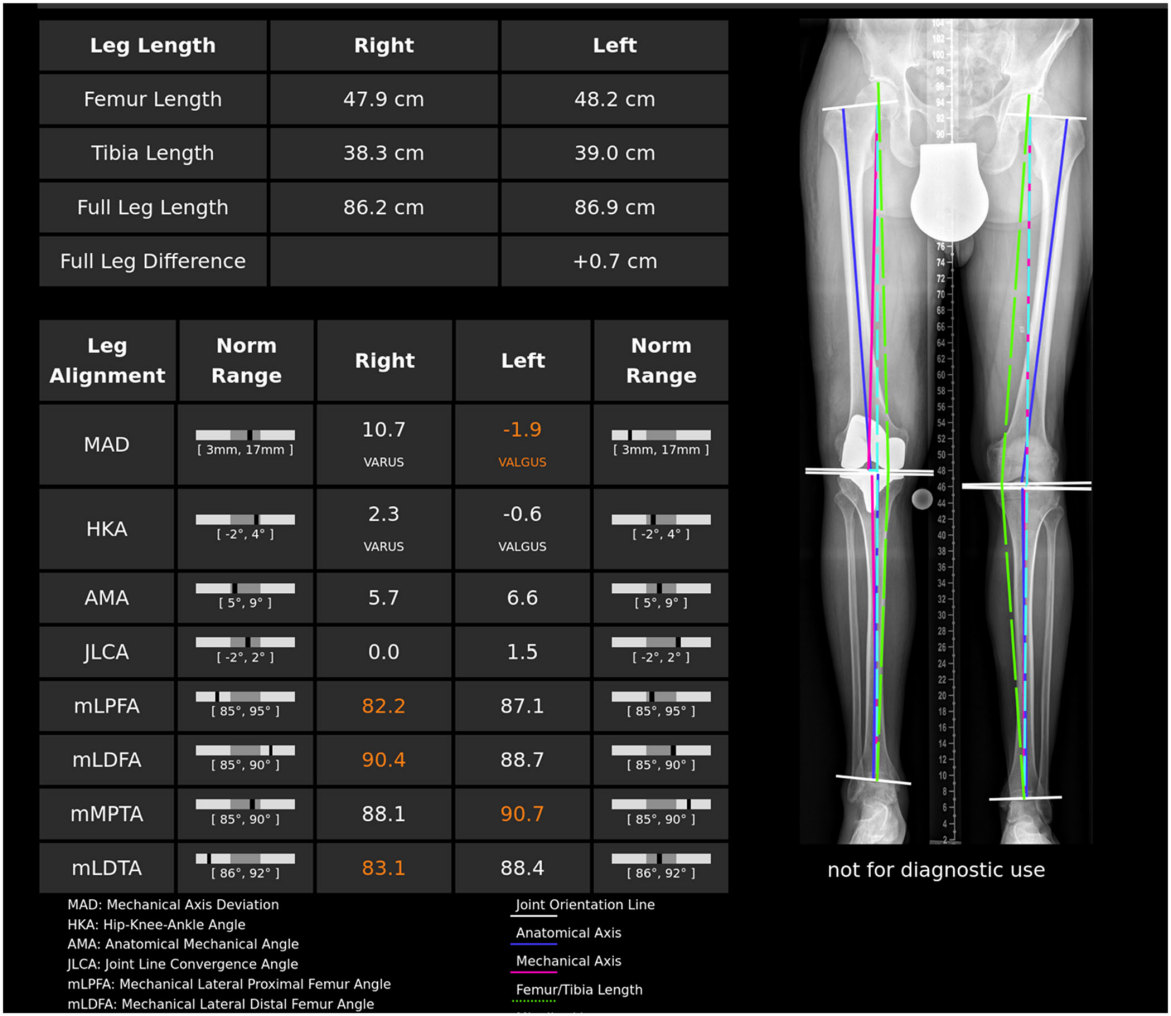
LAMA Artificial intelligence (AI) software for standing leg X‐rays.

AI has shown encouraging performance in accurate quantitative measurements for radiographic measurements, including scoliosis and limb lengths, improving assessment and treatment planning in musculoskeletal care. For scoliosis, an AI model (cobbAngle pro) has demonstrated good accuracy and repeatability in automatically measuring the Cobb angle, eliminating the need for manual measurements by clinicians [23]. Comparative studies have highlighted the superior performance of algorithms trained on both adolescent idiopathic scoliosis and adult spinal deformity cases, with convolutional neural networks achieving intraclass correlation coefficients of 0.973 for major curves in the standing position [17, 27]. In limb length measurement, AI systems have shown exceptional accuracy, with correlation coefficients exceeding 0.99 and mean errors under 1% [21, 38]. Deep learning approaches for bilateral leg length assessments have demonstrated high concordance with radiologists, with intraclass correlation coefficients of 0.979 for whole‐leg lengths, 0.905 for tibial lengths, and 0.979 for femoral lengths [29]. The IB Lab LAMA AI software further enhances measurements on long‐leg radiographs, including cases with hip or knee implants, aiding in identifying alignment deformities and discrepancies (Figure 1) [39]. These AI‐powered tools improve measurement accuracy, reduce inter‐observer variability, standardise assessments across centres, and significantly reduce measurement times—some by up to 87% [1]. Additionally, they offer support in remote settings lacking specialist radiologists [1, 51]. By enhancing accuracy, objectivity, and efficiency, AI tools are advancing the assessment and treatment planning for scoliosis and limb length discrepancies, ultimately enabling better treatment decisions and improved patient care.

Diagnosing osteoarthritis (OA) on knee radiographs, particularly identifying early disease signs is another application for AI modes. A model developed by researchers at the University of Pittsburgh and Carnegie Mellon University detected OA on MRI scans with high accuracy three years before symptom onset [20]. Similarly, a ResNet101‐based AI platform demonstrated high accuracy in distinguishing OA grades, excelling in early‐stage detection [22]. An AI neural network from the University of Jyväskylä matched clinicians' diagnoses of early OA in 87% of cases, and AI‐aided radiographic analysis improved inter‐rater reliability [31, 36]. Advanced methods like 3D transport‐based morphometry further enable the detection of biochemical cartilage changes via MRI [20]. By offering earlier intervention, improved accuracy, expedited screening, and consistent assessments, AI enhances OA diagnosis while complementing clinical expertise [42]. Ongoing research is essential to optimise its role in advancing patient care.

## ETHICAL CONSIDERATIONS

Ethical considerations are critical for the responsible implementation of AI in musculoskeletal imaging, particularly in ensuring patient care and equitable outcomes. Protecting data privacy is paramount, requiring secure storage, robust de‐identification, and adherence to regulations [16]. To mitigate bias, AI must be trained on diverse, representative datasets and regularly assessed for its impact on different demographics [7]. Transparency and explainability are equally vital, as interpretable AI models foster trust by providing clear rationales for their outputs, enabling both clinicians and patients to make informed decisions [13, 34, 35]. Accountability frameworks are necessary to clarify responsibilities for AI‐assisted decisions, emphasising that radiologists and the treating physicians retain ultimate responsibility for patient care [10]. Additionally, ensuring data quality and model efficacy involves rigorous validation and continuous monitoring of AI tools [11]. By addressing these ethical issues, the integration of AI can improve musculoskeletal imaging while safeguarding privacy, promoting fairness, and maintaining trust in clinical decision‐making.

## CONCLUSION

AI is transforming musculoskeletal imaging by enhancing diagnostic accuracy, streamlining workflows, and enabling personalised care. Despite challenges including anatomical complexity, image quality issues, and ethical concerns, advancements in both multimodal and specialized models show immense promise. To fully realise AI's potential, rigorous validation, ethical safeguards, and seamless clinical integration are essential. By fostering collaboration between clinicians and AI tools, the field can achieve improved efficiency, equity, and outcomes, marking a new era in precision medicine.

## AUTHOR CONTRIBUTIONS

All listed authors have contributed substantially to this work: Felix C. Oettl, David Fendrich and Robert Feldt performed literature review. Felix C. Oettl performed primary manuscript preparation. Editing and final manuscript preparation was performed by Bálint Zsidai, Jacob F. Oeding, Michael T. Hirschmann, Matthew J. Kraeutler, Philipp W. Winkler, Pawel Szaro and Kristian Samuelsson. All authors read and approved the final manuscript.

## CONFLICT OF INTEREST STATEMENT

Kristian Samuelsson is a member of the Board of Directors of Getinge AB (publ) and medtech advisor to Carl Bennet AB.

## ETHICS STATEMENT

None declared.

## Data Availability

Data sharing not applicable to this article as no datasets were generated or analysed during the current study.
